# Candidate Glutamatergic Neurons in the Visual System of *Drosophila*

**DOI:** 10.1371/journal.pone.0019472

**Published:** 2011-05-05

**Authors:** Shamprasad Varija Raghu, Alexander Borst

**Affiliations:** Department of Systems and Computational Neurobiology, Max-Planck-Institute of Neurobiology, Martinsried, Germany; Columbia University, United States of America

## Abstract

The visual system of *Drosophila* contains approximately 60,000 neurons that are organized in parallel, retinotopically arranged columns. A large number of these neurons have been characterized in great anatomical detail. However, studies providing direct evidence for synaptic signaling and the neurotransmitter used by individual neurons are relatively sparse. Here we present a first layout of neurons in the *Drosophila* visual system that likely release glutamate as their major neurotransmitter. We identified 33 different types of neurons of the lamina, medulla, lobula and lobula plate. Based on the previous Golgi-staining analysis, the identified neurons are further classified into 16 major subgroups representing lamina monopolar (L), transmedullary (Tm), transmedullary Y (TmY), Y, medulla intrinsic (Mi, Mt, Pm, Dm, Mi Am), bushy T (T), translobula plate (Tlp), lobula intrinsic (Lcn, Lt, Li), lobula plate tangential (LPTCs) and lobula plate intrinsic (LPi) cell types. In addition, we found 11 cell types that were not described by the previous Golgi analysis. This classification of candidate glutamatergic neurons fosters the future neurogenetic dissection of information processing in circuits of the fly visual system.

## Introduction

In flies processing of visual information starts in the repetitively arranged ommatidia of the compound eye. Each ommatidium has its own little lens, a cluster of 8 photoreceptor cells surrounded by support and pigment cells. The photoreceptors send axons into a part of the brain exclusively devoted to image processing called the ‘optic lobe’. In the optic lobe, the visual signals become split and processed in many parallel channels [1], [2], [3], [4], [5]. However, detailed descriptions of individual cell types and neural circuits that process different visual tasks like color, form and motion are not available so far. *Drosophila melanogaster* offers a number of genetic tools suitable to address the connectivity and functional neuroanatomy of identified cell types. Such an analysis in *Drosophila* likely should also shed light on similar neurons in larger fly species as columnar cell types of the optic lobe show a high degree of evolutionarily conservation across dipteran flies [6].

The optic lobe of dipteran flies consists of four neuropiles: lamina, medulla, lobula and lobula plate that host a total of approximately 60,000 cells in *Drosophila*. Many of these neurons were characterized by Golgi-impregnation [1]. The lamina neurons connect to the medulla via the outer chiasm where fibers cross to switch anterior and posterior positions, while retaining their positions along the dorso-ventral axis [1]. The medulla is divided into 10 layers, running orthogonally to the columns, with M1 being the distal most and M10 being the proximal most layer. Most of the columnar neurons in the medulla are well described for two fly species, the house fly *Musca domestica* [2] and the fruit fly *Drosophila melanogaster* [1]. The medulla is connected to the lobula and lobula plate via the inner chiasm. The lobula neuropile can be divided into six layers running perpendicular to the lobula columns [1] and represents a large number of both columnar and non-columnar cell types. Amongst the different neurons in the *Drosophila* lobula plate, the best studied cells are wide-field lobula plate tangential cells (LPTCs) [7], [8], [9]. In *Calliphora vicina*, LPTCS represent a group of about 60 cells per hemisphere, each of which can be identified individually because of its characteristic anatomy and visual response properties [10], [11], [12], [13]. Similar to their counterparts in *Calliphora*, LPTCs in *Drosophila* respond to large-field visual motion as occurring during certain flight maneuvers of the fly [14], [15].

Histochemical analysis suggests that synaptic signaling in the *Drosophila* visual system relies on different neurotransmitter systems: Acetyl Choline [16], [17], [18], [19], [20], GABA [18], [19], [20], [21], glutamate [19], [20], [22], aspartate [19], taurine [23], dopamine [24], [25], serotonin [26], octopamine [27], [28] and histamine [29], [30], [31], [32]. However, establishing a definite relationship between cell types and their used neurotransmitter is notoriously difficult when employing immunolabeling of the whole brain. In order to identify candidate cholinergic neurons in the fly visual system we recently established an anatomical map [33] by employing the promoter for Cha which is specifically active in cholinergic neurons and by restricting its activity to single or few cells by mosaic analysis with repressible cell marker (MARCM). Thereby, we identified 43 different types of cholinergic neurons, 31 of them represent previously described members of 9 subgroups [1]. 12 newly identified cholinergic neurons suggested that the actual number of different neurons per column is higher than previously thought.

Here we extend this study to candidate glutamatergic neurons in the *Drosophila* visual system. We employ the promoter ‘d*vGlut*’ which is specific to glutamatergic neurons [34] and restrict its expression to a few cells using the Flp-out technique [35]. d*vGlut* is the *Drosophila* vesicular glutamate transporter, an integral protein of the synaptic vesicle membrane pumping glutamate into the vesicle lumen [36], [37]. In mammals, three *vGlut* isoforms exists. In *Drosophila*, a single *vGlut* ortholog, *dvGlut*, has been identified [38] and was localized on synaptic vesicle.

We used the line *dvGlut^CNSIII^*-Gal4 that expresses the yeast transcription factor Gal4 under the control of a *dvGlut* promoter fragment [34]. The *dvGlut^CNSIII^*-Gal4 drives expression in about 5,000 neurons in the optic lobe as reported in a recent study [39]. However, the *dvGlut^CNSIII^*-Gal4 expression pattern covers about 60% of all the *dvGlut* immunopositive neurons in the optic lobe. Furthermore, about 20% of the neurons that are labeled by *dvGlut^CNSIII^*-Gal4 expression do not show immunoreactivity to an antibody against the *dvGlut* protein [34]. The *dvGlut^CNSIII^*-Gal4 expression pattern was revealed by crossing it to UAS-mCD8-GFP [40]. Under these conditions a widespread and rather evenly distributed expression of mCD8-GFP was observed in the lamina, medulla, lobula and lobula plate [34]. To restrict mCD8-GFP expression to single or few cells of the complete *dvGlut^CNSIII^*-Gal4 expression pattern, we applied mild heat shocks (37°C) to larvae that in addition carry the transgenes UAS-FRT-CD2*y* ^+^- FRT-mCD8-GFP [35] and a heat-shock inducible flipase (hs-flp). Excision of the *CD2y^+^* stop cassette by the heat shocks at different developmental stages allowed us to identify 33 different types of candidate glutamatergic neurons within the lamina, medulla, lobula and the lobula plate. Neurons were identified based on their structural similarities to the previously described Golgi-stained neurons in *Drosophila* [1]. The identified *dvGlut^CNSIII^*-Gal4 expressing neurons fall into 16 major subgroups representing lamina monopolar (L), transmedullary (Tm), transmedullary Y (TmY), Y, medulla intrinsic (Mi, Mt, Pm, Dm, Mi- Am), bushy T (T), translobula plate (Tlp), lobula intrinsic (Lcn, Lt, Li), lobula plate tangential (LPTCs) and lobula plate intrinsic (LPi) cell types. In addition, 11 cell types are described here that have previously not been reported.

## Materials and Methods

### Fly culture

*Drosophila melanogaster* were grown on standard corn medium at 25°C and 60% humidity. For all experiments flies were kept in 30 ml - vials containing 10 ml food.

### Fly stocks and clonal analysis

We used the Gal4-UAS system to direct gene expression to defined populations of neurons within the *Drosophila* brain [41]. To examine all the Gal4- positive neurons, the *dvGlut^CNSIII^*-Gal4 line (kindly provided by A. Diantonio, Washington University School of Medicine, Missouri) was crossed to a fly line carrying the UAS-mCD8-GFP construct (kindly provided by L. Luo, Stanford University). For single cell analysis, female flies of hs-flp/+; UAS, FRT-CD2*y* ^+^-FRTmCD8:: GFP/+; *dvGlut^CNSIII^*-Gal4/+ genotype were generated. To remove the Flp-out cassette (CD2*y* ^+^), 2–5 days old larvae were exposed to heat shocks (45 to 60 minutes at 37°C in a water bath).

### Immunohistochemistry

Female flies were dissected three to five days after eclosure. Their brains were removed and fixed in 4% paraformaldehyde for 30 minutes at room temperature. Subsequently, the brains were washed for 45–60 minutes in PBT (phosphate buffered saline (pH 7.2) including 1% Triton X-100). For antibody staining, the samples were further incubated in PBT including 2% normal goat serum (Sigma Aldrich, G9023) and primary antibodies (1∶200, overnight at 4°C). Antibodies were removed by several washing steps (5×20 minutes in PBT) and secondary antibodies were added (1∶200, overnight at 4°C). A 5×20 minutes washing protocol (PBT) was followed by final washing steps in PBS (5×20 minutes). The stained brains were mounted in Vectashield (Vector Laboratories, Burlingame) and analyzed by confocal microscopy (see below). The following primary and secondary antibodies were used in the present study: Alexa Fluor 488 rabbit anti-GFP-IgG (A-21311, Molecular Probes), mouse anti-Dlg (Developmental Studies Hybridoma Bank, University of Iowa, Iowa City) and mouse-Alexa Fluor 594 (A-11005, Molecular Probes).

### Microscopy and Data Analysis

Serial optical sections were taken at 0.5 µm intervals with 1024×1024 pixel resolution using confocal microscopes (LEICA TCSNT) and oil-immersion 40×- (n.a. = 1.25) and 63×- (n.a. = 1.4) Plan-Apochromat objectives. In most cases, horizontal sections were taken in the dorsal region of the brain. The individual confocal stacks were analyzed using Image J (NIH, U.S.A) software. The size, contrast and brightness of the resulting images were adjusted with Photoshop® CS (Adobe Systems, San Jose, CA).

## Results

Here we present a large scale clonal analysis using the *dvGlut^CNSIII^*-Gal4 driver line [34] and the Flp-FRT system [35]. Examination of more than 1,000 adult brains allowed us to identify 33 types of different candidate glutamatergic neurons of the lamina, medulla, lobula and lobula plate. Individual cells were identified based on their specific arborization in different strata of the neuropile and position of their cell body as described previously [1]. Images were taken at 0.5 µm thickness and several confocal image stacks were merged to get the complete picture of individual neurons. In the following, we describe the anatomy and characteristic arborization pattern of different candidate glutamatergic neurons.

### Lamina monopolar cells

**Figure 1:**
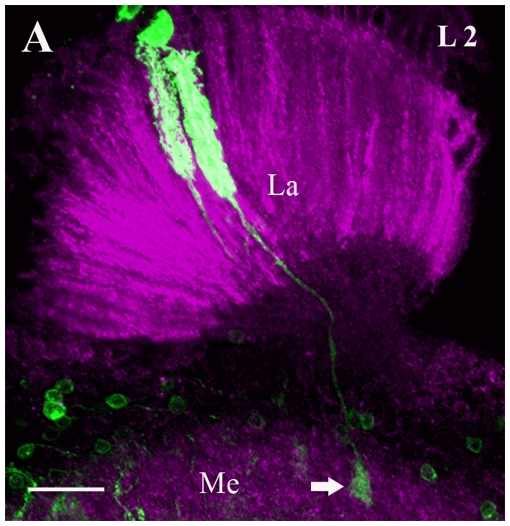
GFP expression in L2 Lamina monopolar cells suggests that L2 neurons are glutamatergic. Two L2 cells were visualized after removal of a stop cassette preceding the cDNA encoding GFP (green). Flip-out of the stop cassette allows Gal4 expressed from the *dvGlut* promoter to induce GFP expression (see methods). The neuropile was labeled using antisera against Dlg, a postsynaptic marker protein (magenta). L2 cells have its cell body in the outer cell body rind of the lamina. The dendritic arborization covers the entire lamina longitudinally and turns into an axon that terminates in layer M2 (arrow) of the medulla. The image represents maximum intensity projections of 30 images. Individual images were taken at every 0.5 µm along the z-axis. La - lamina and Me - medulla. Scale Bar: 20 µm.

The lamina contains five types of lamina monopolar neurons (LI-L5). These neurons differ in their shape and connectivity. In the present analysis, we found L2 monopolar cells (L2) to activate the *dvGlut* promoter (Fig. 1). L2-cells form uniform arrangements of short, radially-directed dendritic branches throughout the depth of the lamina neuropil. Their cell bodies lie in the layer between the basement membrane of the compound eye and the lamina neuropil. In the medulla, L2 cell terminate within the M2 layer. No other lamina monopolar neurons were labeled in the present analysis.

### Medulla Projection Neurons

Medulla projection neurons are the major group of columnar neurons, connecting one or several layers of the medulla to the lobula or lobula and lobula plate. Based on their target region, these neurons are grouped into two major classes: Transmedullary or Tm cells and Transmedullary Y or TmY cells. Tm cells connect different layers of medulla to the lobula where as TmY cells connect the medulla to both the lobula and the lobula plate, bifurcating in the inner chiasm. Both Tm and TmY cells have their cell bodies distal to the medulla neuropile.

**Figure 2:**
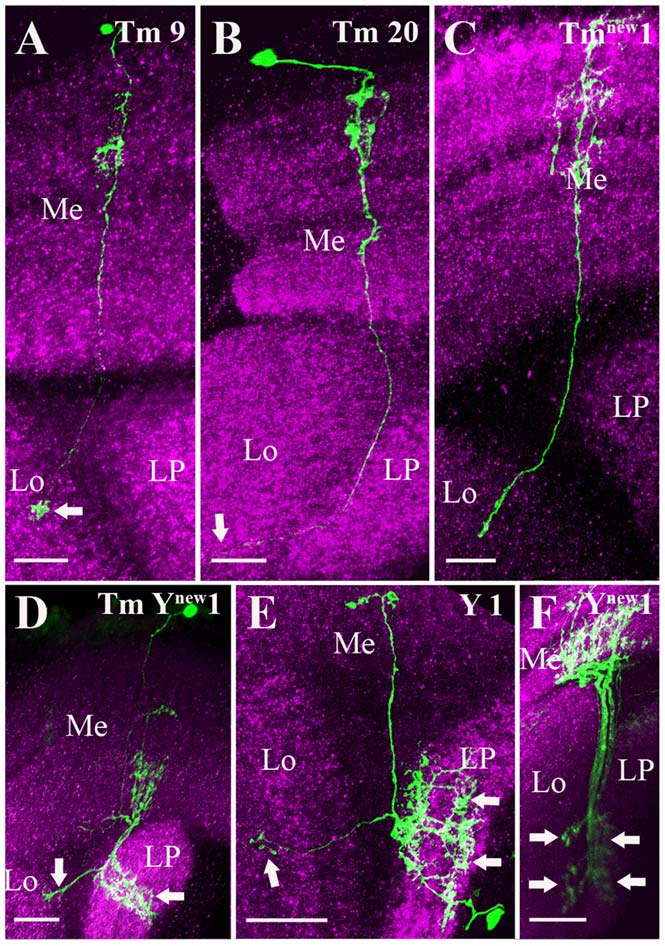
Candidate glutamatergic projection neurons of the fly visual system. Three groups of projection (Transmedullary (Tm), Transmedullary Y (TmY) and Y) neurons were visualized and identified as described in Fig. 1. Tm9 (A), Tm20 (B) and Tm^new^1(C) neurons have their cell bodies distal to the medulla neuropile, wide spread ramifications in one or several layers of the medulla and fine terminal ramifications in the lobula (arrows). TmY^new^1 (D) neuron mainly branches into M5, M8, M9 and M10 layers in the medulla. In the lobula it terminates into inner most layers (arrow) where as in the lobula plate it covers all the four layers (arrow) and cell body lies in the outer chiasm between medulla and lamina. Y1 (E) cell connects M9 layer in the medulla to the lobula and lobula plate. In the lobula Y1 cell terminates in to layer 4 and 5 where as in the lobula plate it covers almost all the layers (arrows). The cell body of Y1 cell lies outside the lobula plate. Y^new^1 (F) cell has arborization in the M8–M10 layers in the medulla and sends branches to innermost layers of both lobula and lobula plate (arrows). Images A to F are maximum intensity projections of 24, 32, 16, 34, 33 and 24 images, respectively. Individual image were taken at every 0.5 µm along the z-axis. Me - medulla, Lo - lobula and LP - lobula plate. Scale bar: 10 µm for images A–C and 20 µm for images D–F.

Heat-shock induced removal of the FRT-flanked Stop cassette labeled two different types of Tm cells Tm9 (Fig. 2A) and Tm20 (Fig. 2B) and new Tm cell (Tm^new^1, Fig. 2C). Tm9 was identified by its arborizations in the medulla layers M2, M3 and M4 and its terminal in the layer 1–2 of the lobula (arrow, Fig. 2A). Tm20 arborizes in medulla layers M1–M4 and M8 and terminates in the layer 5 of the lobula (arrow, Fig. 2B).

In the present analysis none of the previously characterized TmY cells [1] were labeled. However a new cell type (TmY^new^1, Fig. 2D) was identified. It mainly branches within M5, M8, M9 and M10. In the lobula, it terminates within the inner most layer. In the lobula plate it covers all the four layers. Similar to other TmY cells, the cell body of this cell lies in the outer chiasm between the medulla and the lamina. The overall morphology of this cell matches another group of transmedullary cells called Y cells. However, the cell bodies of so far described Y cells lie dorsal to the lobula plate.

We also found two different classes of Y-cells: Y1 (Fig. 2E) and a new Y cell type (Y^new^1, Fig. 2F). Y1 cell (Fig. 2E) connects the medulla layer M9 to the lobula and lobula plate. In the lobula, Y1 terminates within layers 4 and 5, whereas in the lobula plate it covers almost all the layers. The cell body of this cell lies outside the lobula plate. The Y^new^1-cell (Fig. 2F) has arborization in the medulla layers M8–M10 and sends branches to the innermost layers of the both lobula and the lobula plate (arrows).

### Medulla intrinsic cells

Medulla intrinsic cells extend distinct ramifications exclusively with in the medulla, connecting different layers of the medulla to each other. There are several subgroups of medulla intrinsic neurons.

**Figure 3:**
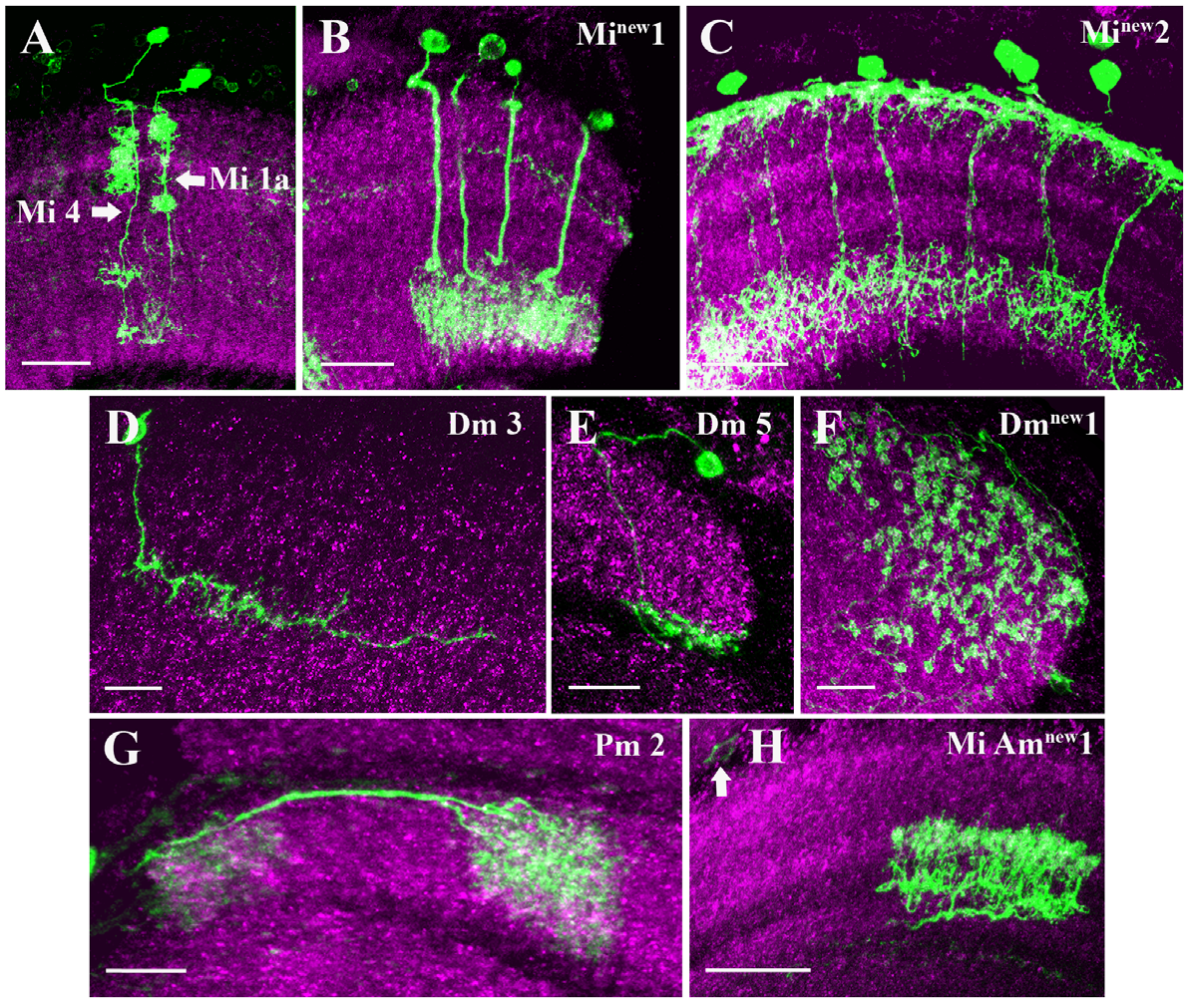
Candidate glutamatergic medulla intrinsic neurons. Medulla intrinsic (Mi), dorsal medulla (Dm), proximal medulla (Pm) and medulla intrinsic amacrine cells (Mi Am) neurons were visualized as described in the legend of Fig. 1. Mi1a (A) projects mainly to M1, M5 and M9–M10 layers in the medulla and in addition have smaller branches between M1 to M5 layers. Mi4 (A) covers layers M1–M5 and M8–M9 in the medulla and connects the distal with the proximal medulla neuropile. Mi^new^1 cell (B) has arborization in layers M8–M9 and cell body in the outer chiasm region between medulla and lamina. Mi^new^2 (C) sends terminals to M1 and M8–M9 layers in the medulla. Dm3 (D) cell extend its processes to medulla layer M2–M3. The cell bodies are found in the outer rind of medulla. Dm5 (E) cell extend its processes mostly near the serpentine layers and connects dorsal and proximal region in the medulla. The cell body of Dm5 cell lies in the outer rind of medulla. Dm^new^1 (F) cell cover mostly the dorsal part of the medulla spreading over layers M1 to M4 and has characteristic blob like protrusion at the terminals. Pm2 (G) cells have their processes in M8–M9 layers in the medulla and their cell bodies are found in the posterior part of the medulla. Mi Am^new^1 (H) cell has arborization in the M5–M7 layers in the medulla. Images A to H are maximum intensity projections of 31, 29, 14, 9, 25, 36, 31 and 44 images, respectively. Individual image were taken at every 0.5 µm along the z-axis. Scale Bar: 20 µm for A–C, 10 µm for D–F and 20 µm for G–H.

The columnar medulla intrinsic (Mi) cells send branches to a minimum of two different layers of the medulla. Golgi analysis revealed 12 types of Mi cells in *Drosophila* [1]. Four types of Mi cells were included in the *dvGlut^CNSIII^*-Gal4 expression pattern: Mi 1a (Fig. 3A), Mi 4 (Fig. 3A) and two new cell types (Mi^new^1, Mi^new^2, Fig. 3B–C). Mi1a projects to M1, M5 and M9–M10, similar to Mi1 cells, but in addition has some smaller branches in the layers between M1 to M5. Mi4 covers layers M1–M5 and M8–M9 and thus connects the distal with the proximal medulla neuropile. Mi^new^1 cells (Fig. 3B) have arborization in layers M8–M9 and their cell bodies in the outer chiasm region between medulla and lamina. Cells with similar morphology have been previously reported in *Musca* [2] and in *Drosophila* [53]. The cell type described in Morante and Desplan [53], has arborizations in M9 and M10, hence called Pm_9–10_. However, the cells described in the present analysis do not branch in layer M10. To our knowledge, such cell types have not yet been described in the *Drosophila* optic lobe. Mi^new^2 (Fig. 3C) sends terminals to M1 and M8–M9. It somewhat resembles the Mi10 cell, but Mi10 cell ramifies in the medulla layer M3 instead of M1.

We also identified three candidate glutamatergic non-columnar neurons of the medulla, the dorsal medulla neurons (Dm): Dm3, Dm5 and Dm^new^1 (Fig. 3D, E, F). Dm3 cells extend their processes to medulla layers M2–M3. The cell bodies are found in the outer rind of medulla. Dm5 cells ramify mostly near the serpentine layers and connect dorsal and proximal region in the medulla. The cell bodies of Dm5 cells (Fig. 3E) are found in the outer rind of medulla. Dm^new^1 (Fig. 3F) covers mostly the dorsal part of the medulla spreading over layers M1 to M4. These cells have characteristic blob-like protrusion at the terminals.

The proximal medulla neurons (Pm) are also non-columnar medulla neurons. Pm cells ramify in the proximal region of the medulla. In the present study, Pm2 cells (Fig. 3G) were labeled. Pm2 cells branch within layers M8–M9, their cell bodies are found in the posterior part of the medulla. The Mi Am^new^1 (Fig. 3H) cell identified in the present analysis arborizes mainly within layers M5–M7. The cell bodies lie in the posterior part of the medulla (arrow, Fig. 3H).

### Medulla Tangential cells

**Figure 4:**
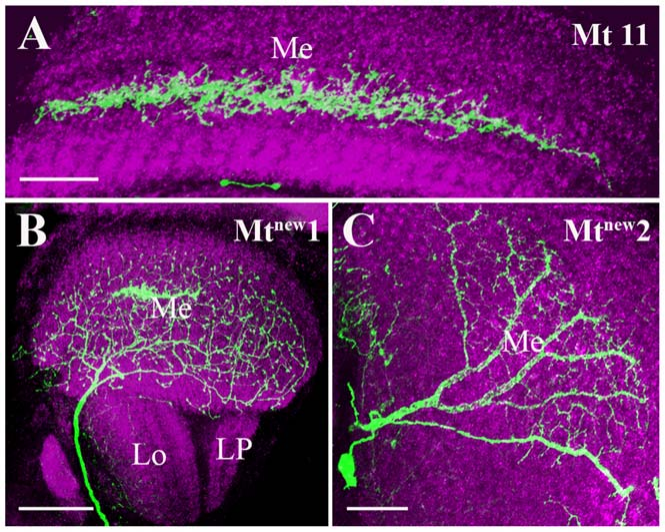
Candidate glutamatergic Medulla tangential (Mt) cells. The cells are visualized as described in the legend of Fig. 1. Mt cells run across the medulla, mostly in parallel and close to the serpentine layer. The cell bodies of most Mt neurons are situated anterior to the medulla neuropile. Mt11 (A) cell covers almost complete visual field and run across the serpentine layer. Mt^new^1 (B) also spread across complete medulla neuropile, while Mt^new^2 cell (C) covers only part of the visual field. In A and B, horizontal sections were taken in the dorsal region of the brain. In C, frontal sections were taken from the posterior side of the brain. Images A to C are maximum intensity projections of 11, 36 and 17 images, respectively. Individual image were taken at every 0.5 µm along the z-axis. Me - medulla, Lo - lobula and LP - lobula plate. Scale Bar: 20 µm for A, 40 µm for B and 20 µm for C.

Medulla tangential (Mt) cells represent a group of about 15 wide field neurons that run across the medulla, mostly in parallel and close to the serpentine layer. The cell bodies of most medulla tangential neurons are situated anterior to the medulla neuropile [1]. In the present analysis we visualized 3 different Mt cells. Mt11 (Fig. 4A) cells have been previously described [1] and cover almost the complete visual field. The new cell type Mt^new^1 (Fig. 4B) also spreads across the complete medulla neuropile, while Mt^new^2 cell (Fig. 4C) covers only part of the visual field.

### Bushy T-cells

**Figure 5:**
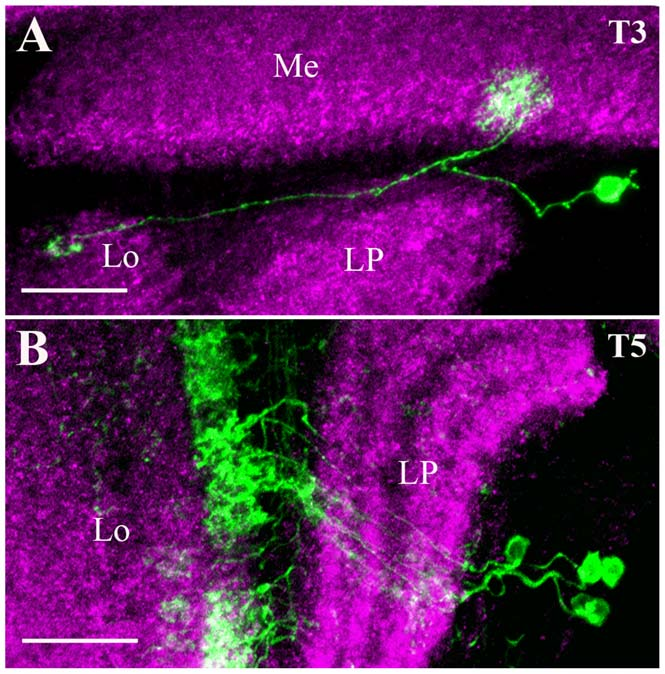
Candidate glutamatergic T Bushy (T) cells. T3 (A) and T5 (B) cells were visualized and identified as described in the legend of Fig. 1. The cell body of T3 (A) cell clustered posterior in the area between the medulla and lobula plate. In the medulla, the T3 neurons arborize within single columns of the proximal medulla, by passing the innermost layer and finally terminate in to layer 3 in the lobula. The cell bodies of T5 (B) cells lie in the rind of the lobula plate. T5 cells connect innermost of layer of the lobula to the lobula plate neuropile. Images A and B are the maximum intensity projections of 35 and 10 images. Individual image were taken at every 0.5 µm along the z-axis. Me - medulla, Lo - lobula and LP - lobula plate. Scale Bar: 20 µm.

The cell bodies of T3 cells (Fig. 5A) cluster in the area between the medulla and lobula plate. In the medulla, T3 neurons arborize within a single column of the proximal medulla, in layer M9 and terminate within the 3^rd^ layer in the lobula.

The cell bodies of T5 cells (Fig. 5B) lie posterior of the lobula plate. T5 cells connect the innermost layer of the lobula to the lobula plate neuropile. Fischbach and Dittrich [1], reported the existence of 4 variants of T5 neurons, based on their branching pattern in the four layers in the lobula plate. In the present study, we found GFP labeling in three variants of T5-cells judged by the position of their axon terminal in layer 1, 3 or 4 of the lobula plate.

### Translobula plate cells

**Figure 6:**
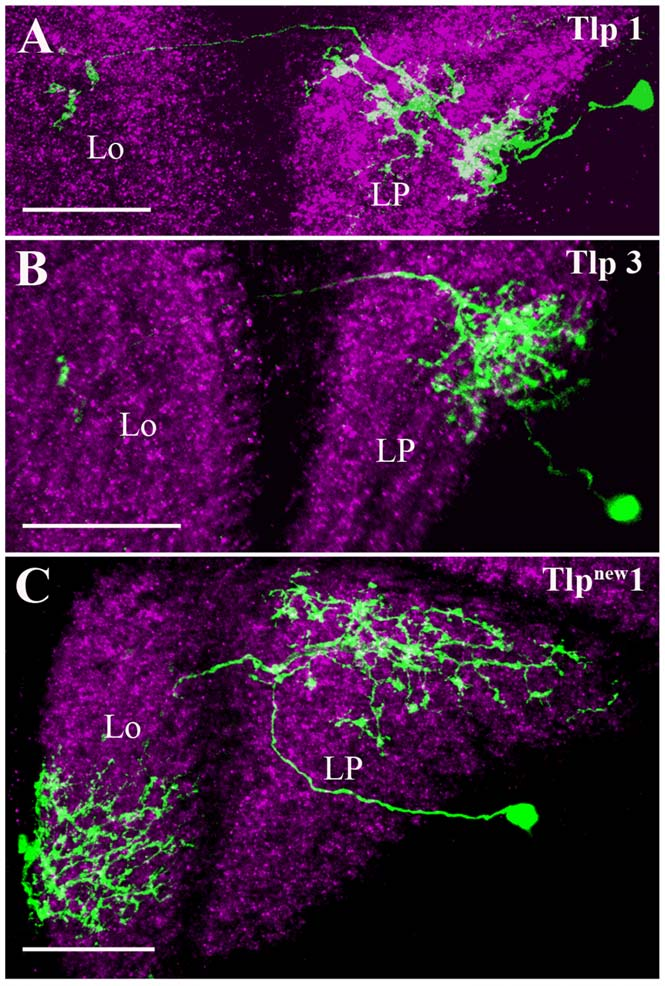
Candidate glutamatergic Translobula plate (Tlp) cells. Tlp cells are identified and visualized as described in the legend of Fig. 1. Tlp cells connect the lobula with the lobula plate. Tlp1 (A) has its processes in layer 5 of the lobula and all the four layers in the lobula plate. Tlp3 (B) has its processes in layer 4 of the lobula and layer 2–4 in the lobula plate. Tlp^new^1 (C) sends processes into layer 4, 5 and 6 in the lobula. In the lobula plate Tlp^new^1 processes cover all the four layers. Images A to C are the maximum intensity projections of 17, 55 and 29 images, respectively. Individual image were taken at every 0.5 µm along the z-axis. Lo - Lobula and LP - Lobula Plate. Scale Bar: 20 µm.

Translobula plate (Tlp) cells connect the lobula to the lobula plate. In *Drosophila*, 5 different types of Tlp cells have been described based on their arborization pattern in the lobula and the lobula plate [1]. Here we identified three different types of Tlp cells included in the *dvGlut^CNSIII^*-Gal4 expression pattern: Tlp1 (Fig. 6A), Tlp3 (Fig. 6B) and Tlp^new^1 (Fig. 6C). Tlp1 (Fig. 6A) has its processes in layer 5 of the lobula and all the four layers in the lobula plate, where as Tlp3 (Fig. 6B) has its processes in layer 4 of the lobula and layer 2–4 in the lobula plate. Tlp^new^1 (Fig. 6C) sends processes into layer 4, 5 and 6 in the lobula and all 4 layers in the lobula plate.

### Columnar, tangential and intrinsic cells of the lobula

**Figure 7:**
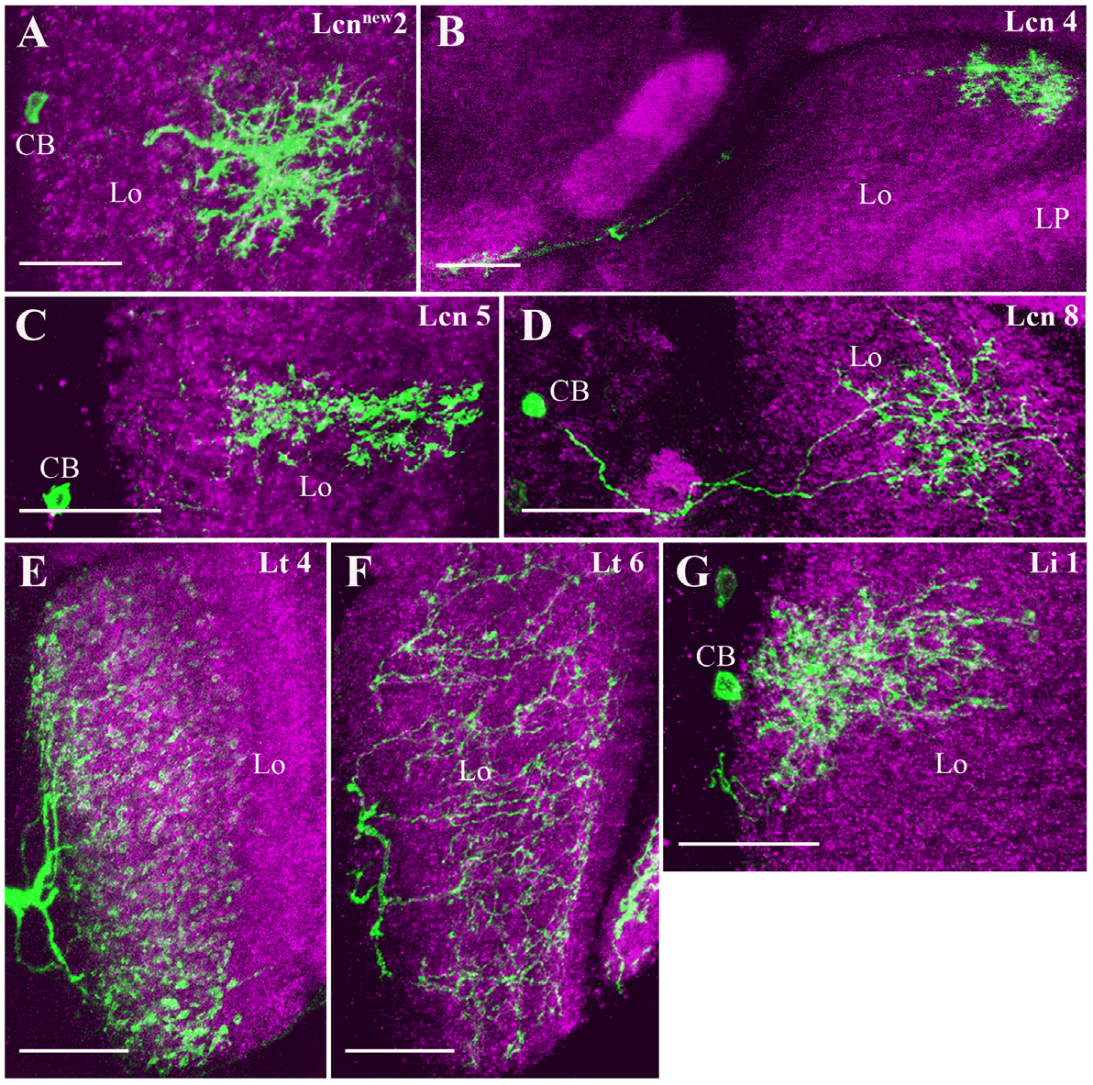
Candidate glutamatergic columnar, tangential and intrinsic cells of the lobula. Lobula columnar (Lcn) cells branch within different layers of the lobula, but mostly spare the most posterior layer, adjacent to the lobula plate, where T5 cells ramify. Putative axons of different Lcn cells are bundled together and project into the central brain. Lcn^new^2 (A) arborizes into layers 3–5 in the lobula. Lcn4 (B), Lcn5 (C) and Lcn8 (D) cover lobula layers 2–4, 4–6 and 4–5 respectively. Lobula tangential (Lt) cells mostly cover different layers within in the lobula. Lt4 (E) sends branches into layer 5 and 6 in the lobula where as Lt6 (F) covers layer 2–5 in the lobula. The putative axons of different Lt cells projects to the different region in the central brain. The cell bodies of Lt cells are located right outside the lobula. Lobula intrinsic (Li) cells too show stratifications within the specific layers of the lobula. These cells differ from Lcn and Lt cells in having relatively short distance axons terminating right outside the lobula. Li1 (G) cell sends arborization into layers 4–6 in the lobula. Images A to G are the maximum intensity projections of 47, 17, 27, 45, 52, 21 and 21 images, respectively. Individual image were taken at every 0.5 µm along the z-axis. The cells are identified and visualized as described in the legend of Fig. 1. Lo - Lobula, LP - Lobula Plate and CB- Cell Body. Scale Bar: 20 µm.

We found three groups of cells in the lobula to be included in the *dvGlut* expression pattern: Lobula columnar (Lcn), lobula tangential (Lt) and lobula intrinsic (Li) cells. Lcn cells branch within different layers of the lobula, but mostly spare the most posterior layer, adjacent to the lobula plate, where T5 cells ramify [1]. Putative axons of different Lcn cells are bundled together and project into the central brain. Lcn^new^2 (Fig. 7A) arborize into layers 3–5 in the lobula like other Lcn2 cells, however differ slightly in their fine branching patters. Lcn4 (Fig. 7B), Lcn5 (Fig. 7C) and Lcn8 (Fig. 7D) cover lobula layers 2–4, 4–6 and 4–5 respectively.

Different tangential neurons that show stratifications within specific layers of the lobula were previously described [1]. In our analysis Lt4 (Fig. 7E) and Lt6 (Fig. 7F) were identified. Lt4 sends branches into layers 5 and 6 of the lobula whereas Lt6 covers lobula layer 2–5. The putative axons of different Lt cells project to different regions in the central brain [1]. The cell bodies of these cells are located right next to the lobula.

Lobula intrinsic (Li) cells show stratifications within specific layers of the lobula. These cells differ from Lcn and Lt cells in having relatively short axons terminating outside the lobula. In contrast to the medulla, the lobula does not contain many intrinsic (Li) neurons [1]. Here, we found one cell type resembling Li1 (Fig. 7G) whose arborizations cover layers 4–6 of the lobula.

### Lobula Plate Tangential cells

**Figure 8:**
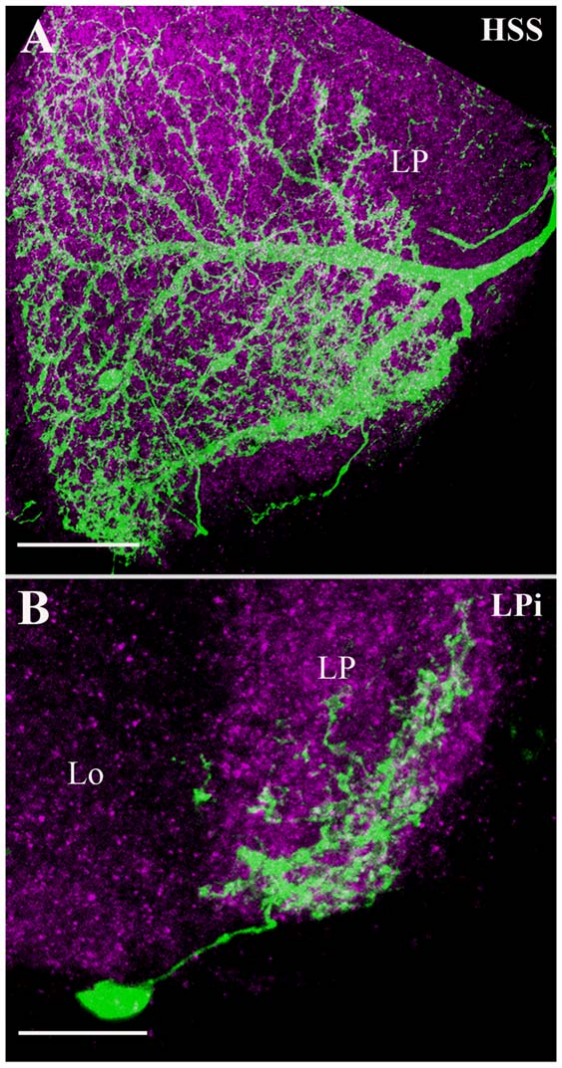
Candidate glutamatergic lobula plate intrinsic cells. Two groups of lobula plate intrinsic cells are labeled: Horizontally Sensitive South (HSS) and lobula Plate intrinsic (LPi) cells. HSS (A) cell represent one of the lobula plate tangential cells (LPTCs) that respond to large-field visual motion and extend its wide spread dendrites in the most ventral part of the lobula plate. LPi (B) cell cover layers 2–4 in the lobula plate. The cell body is located right outside the lobula plate. The cells are identified and visualized as described in the legend of Fig. 1. Images A and B are the maximum intensity projections of 52 and 14 images. Individual image were taken at every 0.5 µm along the z-axis. Lo – Lobula and LP - Lobula Plate. Scale Bar: 20 µm for A and 10 µm for B.

Lobula plate tangential cells (LPTCs) extend their wide spread dendrites in large parts of lobula plate and they respond to large-field visual motion. Within this group of cells, the cells of the horizontal system (HS-cells) comprise three identified neurons, the northern HSN-cell, the equatorial HSE-cell and the southern HSS-cell. Their axons travel medially and terminate in the protocerebrum near the esophagus. In our analysis, we found only the HSS-cell to be contained in the *dvGlut* expression pattern (Fig. 8A). It is possible that HSN and HSE are not glutamatergic or that they have escaped our analysis due to difficulties in generating appropriate clones by the Flp-FRT method. A detailed analysis of the response properties of HSS cells in *Drosophila* has been carried out recently [15].

### Lobula Plate intrinsic cells

Similar to the lobula, there are only few intrinsic (LPi) cells found within the lobula plate [1]. We found one Lpi-cell to be included in the *dvGlut* expression pattern (Fig. 8B). It covers layers 2–4 of the lobula plate. The cell body is located right outside the lobula plate.

**Figure 9:**
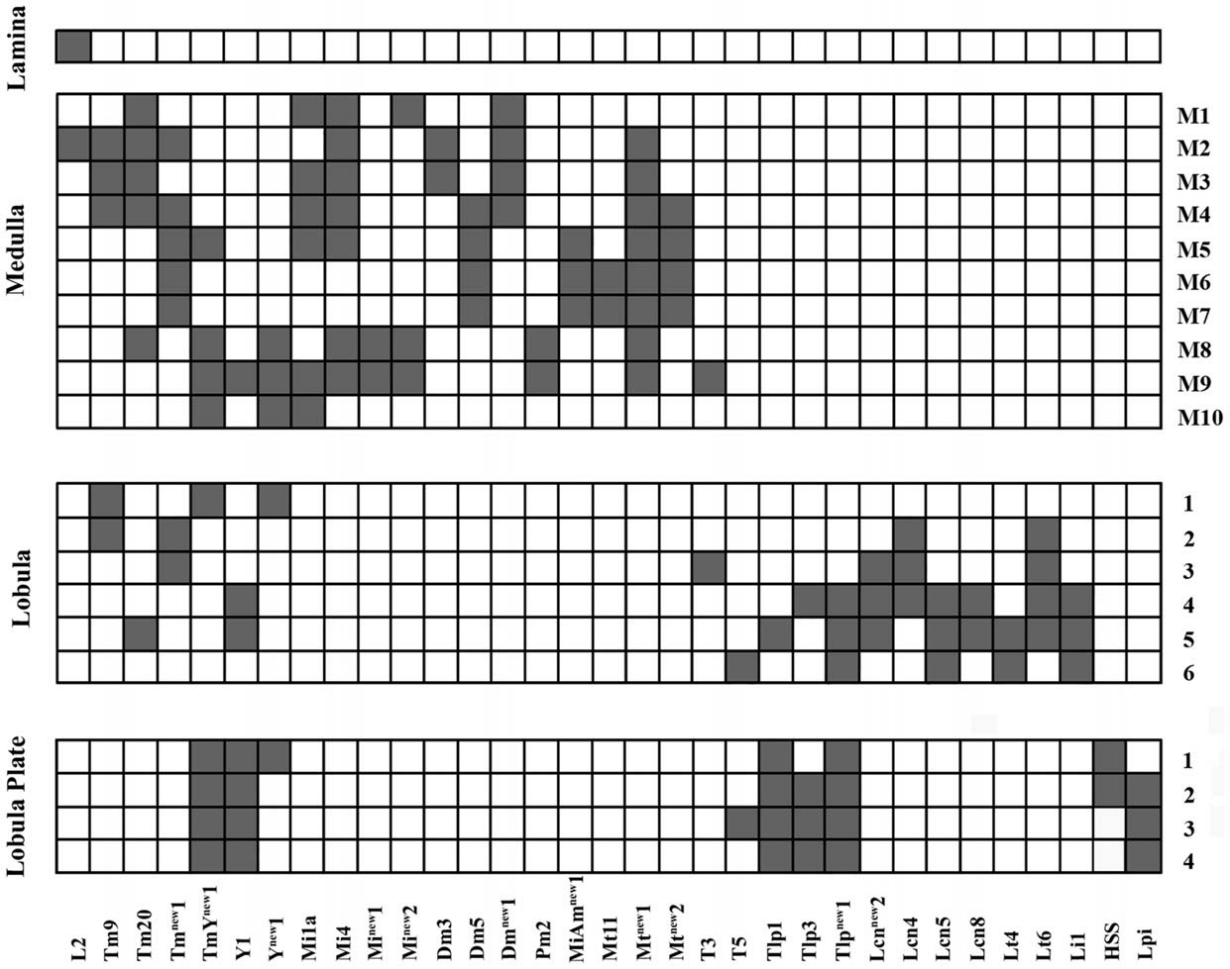
Schematic diagram of the different neuropile layers occupied by the processes of all candidate glutamatergic neuronal types identified. The medulla is subdivided in to 10 layers, the lobula into 6 layers and the lobula plate into 4 layers [1]. Here the shaded boxes indicate the layers in the lamina, medulla, lobula and lobula plate where each neuron ramifies.

An overview of all the different neurons contained within the *dvGlut^CNSIII^*-Gal4 expression pattern is given in Fig. 9. Here, the shaded boxes indicate the layers in which each neuron ramifies within the lamina, medulla, lobula and lobula plate.

## Discussion

Identification and mapping of the neurotransmitter phenotype of individual neurons in the visual system of *Drosophila* is an important step towards interpreting their functional role in defined neural circuits. Our previous analysis identified 43 different types of cholinergic neurons falling into different subgroups [33]. In the present study, we found various candidate glutamatergic neurons in the lamina, medulla, lobula and lobula plate neuropil by combining the *dvGlut^CNSIII^*-Gal4 construct and the Flp-FRT system.

### Genetic tools to address the structural map of candidate glutamatergic neurons

Positively identifying a neuron as glutamatergic is notoriously difficult. This is first of all due to the fact that glutamate is an amino-acid used as a protein precursor as well as an intermediary metabolite. Glutamate is, therefore, found in virtually all cells and not just in those neurons that use glutamate as a neurotransmitter [42]. In addition, nerve terminals that release GABA may contain high levels of cytosolic glutamate because glutamate serves as a substrate in the synthesis of GABA by the decarboxylase reaction [34]. For these reasons, a positive immunolabeling for glutamate provides little insight as to whether a neuron uses glutamate as transmitter or not. As a more specific indicator for glutamatergic neurons in *Drosophila*, Daniel et al [34] used the promoter region of the vesicular glutamate transporter *dvGlut* to label glutamatergic neurons. However, as reported [34], expression of the *dvGlut^CNSIII^*-Gal4 transgene does not cover all neurons that become recognized by an anti-dvGlut antibody. In addition, some cell types included in the *dvGlut^CNSIII^*-Gal4 expression pattern might have escaped our analysis presented here due to difficulties in generating appropriate clones by Flp-FRT method. For these two reasons, our results certainly do not represent a complete list of all glutamatergic neurons in the *Drosophila* optic lobe. Another concern is the ectopic expression of *dvGlut^CNSIII^*-Gal4 in about 20% of the cells in the optic lobe [34]. Therefore, our analysis will not provide with 100% certainty that all neurons described here are indeed glutamatergic. Given these qualifications concerning the completeness and specificity of labeling glutamatergic neurons via the *dvGlut^CNSIII^*-Gal4 construct, the transgenic approach in the present analysis can only provide the first step towards a detailed map of glutamatergic neurons in the visual system of *Drosophila*. Further experiments combining electrophysiological recordings with pharmacological block on each of the cell types will be necessary to unambiguously determine their glutamatergic phenotype.

### Glutamate as a neurotransmitter in the *Drosophila* nervous system

Glutamate has been known for long to be the excitatory transmitter of motor neurons in insects [43], [44]. Thus, the role of glutamate has been studied extensively at the neuromuscular junction, in particular detail in *Drosophila* [45]. Since the neuromuscular junction is large and well accessible to morphological and functional studies throughout development, it is meanwhile well characterized. In contrast, central neuronal synapses of *Drosophila* are comparatively small, complex in number and organization, less accessible to detailed electrophysiological studies and for all these reasons less well characterized. Investigations of the glutamatergic network in the *Drosophila* CNS were largely based on immunohistochemistry and in situ hybridization of different glutamate receptors [46]. These studies showed expression of the glutamate receptor DGluR-IB in defined regions of the adult CNS including accessory medulla, subesophageal ganglia and the outer medulla. In addition, the metabotrobic glutamate receptors DmGluRA has been shown by antibody staining to be present in the optic lobe, antennal lobe, central complex and the median bundle [47]. However electrophysiological studies to reveal the functional role of glutamate receptor subunits in the *Drosophila* CNS are largely missing.

In addition to excitatory glutamate receptors, also glutamate-gated chloride channels exist. These are members of the ligand-gated ion channel superfamily and were first identified in arthropods as extrajunctional glutamate receptors [48]. Two subunits, Glucl-α and Glucl-β of the *C. elegans g*lutamate-gated chloride channel have been cloned [49]. *Drosophila* glutamate chloride channel, DrosGlucl-α shares nearly 48% amino acids and 60% nucleotide identity with the C. elegans Glucl channels. Functional expression of *Drosophila* glutamate-gated chloride channel *in Xenopus* oocytes and the following electrophysiological recordings revealed a glutamate-gated chloride current and demonstrated the inhibitory response of glutamate [49]. However, the physiological relevance of the glutamate-gated chloride channels remains unknown. Nevertheless, the above findings suggest both an excitatory and an inhibitory role for glutamate as a neurotransmitter in the *Drosophila* CNS depending on the postsynaptic glutamate receptor.

### Candidate glutamatergic neurons in the optic lobe of *Drosophila:* Comparison with previous studies

In the fly lamina, immunohistochemical analysis suggested that glutamate signaling is performed at two main sites, the large monopolar cells and the amacrine neuron [20]. In *Drosophila*, only L1 and L2 were detected as glutamatergic in comparison to immunolabeling of L1–L3 in *Musca* and *Calliphora* [19], [50]. Our analysis provides further evidence that L2 cells are indeed glutamatergic (Fig. 1). However, the Cha-promoter has also been shown to be active in L2 neurons, indicative for additional use of Acetyl Choline [51]. Whether this implies a co-release of ACh and glutamate in L2-cells needs to be tested in future experiments. This will be all the more interesting since electrophysiological recordings from motion-sensitive lobula plate tangential cells during transgenetic block of synaptic transmission in L1 and L2 cells have recently demonstrated that L1 and L2 provide the main input to the motion vision system with the L1 pathway being selective for brightness increments and the L2 pathway for brightness decrements [5], [52].

Among the transmedullary neurons we found the Tm9 (Fig. 2A), Tm20 (Fig. 2B) and the Y1 (Fig. 2E) cell to be contained in the *dvGlut* expression pattern. Previous reports suggested that Tm9 are either cholinergic [51] or GABAergic [19]. This raises the possibility of mixed populations of Tm9 cells with different transmitter phenotype or co-release of different neurotransmitters by Tm9 cells. The same applies to the Tm20 cells which have been suggested to be cholinergic [51]. However, further physiological evidences are necessary to prove such co-release of two different neurotransmitters in Tm9 and Tm20 cells. As suggested by Morante and Desplan [53], Y1 cell are likely to be involved in color vision.

Most of the candidate glutamatergic intrinsic neurons of the medulla (Fig. 3A–H) and the lobula complex (Fig. 4A–C, 7A–G and 8A–B) identified in the present study have rather wide spread ramifications that exceed the borders of a single column. Thus, they provide the anatomical basis for lateral intercolumnar interactions such as required for visual motion detection. Mi1 cells have indeed been proposed to be part of the motion detection circuits possibly linking the L1 output to the T4 input [54]. Mi1a cells (Fig. 3A) project to medulla layers M1, M5 and M9–M10, similar to Mi1 cells [1]. However, their additional small branches in the layers between M1 to M5 let us classify them as subtypes of Mi1 cells. Interestingly, Mi1 cells have recently been shown to be ChAT immunopositive, indicating a possible cholinergic phenotype for Mi1 [55]. T5 cells (Fig. 5B) have been proposed to provide major input signals to the motion-sensitive lobula plate tangential cells [54]. This proposal is based on following evidence: T5 cells exist in four different subtypes per column, each ramifying in one of the four different layers of the lobula plate [1]. The same four layers were labeled by activity dependent uptake of 2-deoxyglucose during visual motion presentation [56]. Furthermore, T5-cells have been reported to respond to visual motion in a directionally selective way [57]. In our study, we found a mixed population of T5 cells ramifying into different layers of the lobula plate. A previous immunolabeling study suggested T5-cells in *Apis mellifera* and *Periplaneta americana* to use the transmitter Aspartate [19].

HSS cells (Fig. 8A) have wide spread dendrites covering ventral part of the lobula plate. Their axon travels medially terminating in the protocerebrum near the esophagus. The response properties of the three tangential cells of the horizontal system, including the HSS cell, have been described in exhaustive detail in large fly species [10], [11], [58], [59], [60] as well as in *Drosophila* [15]. These findings demonstrate that HS cells in both species are coupled to each other, either directly [15] or indirectly [61], and respond to large-field motion in a similar way. They finally convey this information onto descending neurons in the protocerebral region, which ultimately control motor neurons for flight and head movements [62], [63], [64], [65], [66], [67]. The glutamatergic phenotype of HS cells was previously reported in *Apis mellifera* and *Periplaneta americana* [19]. Our analysis provides evidence that HSS cells are glutamatergic in flies as well.

All the three Tlp cells (Fig. 6A–C) labeled in the present analysis connect the lobula to the lobula plate. In *Drosophila*, 5 different types of Tlp cells have been previously reported [1]. Tlp2 and Tlp3 cells are cholinergic [33]. The present analysis suggests that Tlp1 (Fig. 6A), Tlp3 (Fig. 6B) and a new Tlp (Fig. 6C) cells might use glutamate as a transmitter.

### Conclusion

In combination with previous data [33], our result presented above has increased the number of neurons in the visual system of *Drosophila* for which a putative neurotransmitter phenotype has now been identified. Interestingly, some of the visual neurons show a mixed cholinergic [33] and glutamatergic phenotype. In the vertebrate retina, starburst amacrine cells (SACs) release the two fast acting neurotransmitters acetylcholine (ACh) and γ-aminobutyric acid (GABA) [68], [69], [70], demonstrating that two neurotransmitters with opposing action can be release from a single neuron. Such a co-transmission of different neurotransmitters might apply to insect visual neurons as well. Future studies using Split-Gal4 approach [71] of promoter driven expression for two different genes of interest in a specific cell population will help to further elucidate this issue. Finally, the identification of new cell types here suggests that the previous Golgi analysis [1], [2] was not saturating indicating that there exist more cells in the *Drosophila* optic lobe than was previously thought.
